# Three-Dimensional Printing of Red Algae Biopolymers: Effect of Locust Bean Gum on Rheology and Processability

**DOI:** 10.3390/gels10030166

**Published:** 2024-02-23

**Authors:** Sónia Oliveira, Isabel Sousa, Anabela Raymundo, Carlos Bengoechea

**Affiliations:** 1 LEAF—Linking Landscape, Environment, Agriculture and Food—Research Center, Associated Laboratory TERRA, Instituto Superior de Agronomia, Universidade de Lisboa, Tapada da Ajuda, 1349-017 Lisboa, Portugal; soliveira@isa.ulisboa.pt (S.O.); isabelsousa@isa.ulisboa.pt (I.S.); anabraymundo@isa.ulisboa.pt (A.R.); 2 Departamento de Ingeniería Química, Escuela Politécnica Superior, Universidad de Sevilla, 41012 Sevilla, Spain

**Keywords:** seaweeds, gels, locust bean gum, hydrocolloids, green extraction, 3D food printing, rheology

## Abstract

Seaweeds, rich in high-value polysaccharides with thickening/gelling properties (e.g., agar, carrageenan, and alginate), are extensively used in the food industry for texture customization and enhancement. However, conventional extraction methods for these hydrocolloids often involve potentially hazardous chemicals and long extraction times. In this study, three red seaweed species (*Chondrus crispus*, *Gelidium Corneum*, and *Gracilaria gracilis*) commercialized as food ingredients by local companies were chosen for their native gelling biopolymers, which were extracted using water-based methodologies (i.e., (1) hydration at room temperature; (2) stirring at 90 °C; and (3) centrifugation at 40 °C) for production of sustainable food gels. The potential use of these extracts as bioinks was assessed employing an extrusion-based 3D printer. The present work aimed to study the gelation process, taken place during printing, and assess the effectiveness of the selected green extraction method in producing gels. To improve the definition of the printed gel, two critical printing parameters were investigated: the addition of locust bean gum (LBG) at different concentrations (0, 0.5, 1, 1.5, 2, and 2.5%) and printing temperature (30, 40, 60, and 80 °C). Rheological results from a controlled-stress rheometer indicated that gels derived from *G. corneum* and *G. gracilis* exhibited a lower gel strength (lower G′ and G″) and excessive material spreading during deposition (lower viscosity) than *C. crispus*. Thus, G′ was around 5 and 70 times higher for *C. crispus* gels than for *G. corneum* and *G. gracilis*, respectively. When increasing LBG concentration (0.5 to 2.5% *w*/*w*) and lowering the printing temperature (80 to 30 °C), an enhanced gel matrix definition for *G. corneum* and *G. gracilis* gels was found. In contrast, gels from *C. crispus* demonstrated greater stability and were less influenced by these parameters, showcasing the potential of the seaweed to develop sustainable clean label food gels. Eventually, these results highlight the feasibility of using algal-based extracts obtained through a green procedure as bioinks where LBG was employed as a synergic ingredient.

## 1. Introduction

Seaweeds, also known as macroalgae, are a group of macroscopic multicellular species, traditionally classified into Phaeophyta (brown), Rhodophyta (red), and Chlorophyta (green), based on their pigmentation. Carbohydrates represent the major component of the biomass of seaweeds, varying between 25 and 50% (green), 30 and 60% (red), and 30 and 50% (brown) dry weight (DW) [1]. Phycocolloids are large molecules present in intercellular spaces in algae, conferring consistency/flexibility to their cell walls [2]. Phycocolloids in red algae (Rhodophyta) are floridean starch and floridoside, which are similar to general starch. The major polysaccharide constituent of red algae are galactans, mostly represented by carrageenan (up to 75% DW) and agar (up to 52% DW) [3]. Industrially, commercial carrageenans are mainly extracted from *Chondrus* spp., *Eucheuma* spp., and *Kappaphycus* spp. [3,4]. However, seaweeds produce hybrid structures of the kappa, iota, and lambda forms of carrageenans, which are composed D-galactopyranose residues bonded by regularly alternating α-(1 → 3) and β-(1 → 4) bonds [5]. Kappa (κ)-carrageenan has one sulfate ester, while iota (ι)- and lambda (λ)- carrageenan contain two and three sulfates per dimer, respectively. Their ability to act as a gelling or thickening agent is dependent on the presence of the anhydro-galactose bridge of the 4-linked galactose residue, crucial for the formation of the helical structure and, consequently, the ability to form a gel. While both the κ-carrageenan and ι-carrageenan share the ability to form gels, they distinguish themselves by producing strong and brittle gels for the κ-type and soft gels for the ι-type. The λ type, usually only described as a thickening agent, has been proved to form gels based on a trivalent cation complexation [6]. Commercial carrageenans find extensive application as additives in the pharmaceutical, cosmetic, and food industries for enhancing and customizing the texture of functional food products, showcasing its phycocolloids relevance from a boasting market size of 872 million USD in 2022 [7]. Commercial agar is mainly extracted from *Gracilaria* spp. (53%) and *Gelidium* spp. (44%) [8,9]. Agar presents unique physicochemical properties, acting as a gelling, thickening, and stabilizing agent, also exhibiting notable biodegradability and a substantial water-holding capacity. Its applications include the food, pharmaceutical, cosmetic, biotechnological, and biomedical sectors, presenting a notable market size valued at 264 million USD in 2022 [10]. Agar is mainly composed of agarose and agaropectin, the former being the gel-forming component, with a linear chain of repeating units of (1,3)-linked-β-D-galactose and (1,4)-linked-3,6-anhydro-α-L-galactose [11]. The gelling ability of agar is hindered by the substitution of the hydroxyl groups of l-galactose by sulphate esters, methyl ethers, or pyruvate acid ketals.

The quality of phycocolloids is industrially valued by its yield, gel strength, and purity. Gel properties of phycocolloids are usually species-dependent and dictated by the environmental conditions and extraction and isolation methods of the polysaccharides. Conventional extraction methods generally include a washing step followed by pre-treatments (employing alkali (e.g., NaOH) or acidified water) and a final hot-water extraction [3]. Alkali treatment has commonly been carried out due to the reportedly weak gel properties of the gels extracted with hot water [12]. However, these multi-step methods are time-, energy-, water-, and solvent-consuming, generating large amounts of wastewater. Therefore, the search for alternative polysaccharide extraction methods that are eco-friendlier, due to the absence of a strong alkali, and predictably cheaper, as fewer steps are included in the extraction process, has boosted over increasing ecological concerns [11,13]. These include microwave-, ultrasound-, and enzyme-assisted extraction, which are relatively environment-friendly technologies but still rather limited to the laboratory scale [14]. Finding adequate operational conditions to adjust the mechanical properties of the formed gels is crucial for the industrial scale-up process.

Polymers extracted from algae have proven to be valid bioinks with the properties required for 3D printing. As those properties are largely influenced by the extraction technique employed, when mechanical features of algal polysaccharides are poor, either mixing with other polymers or chemical modification can be considered to make them suitable for 3D printing [15,16]. Also, the examination of the rheological properties of the bio-inks developed is of paramount importance, as the extrudability, shape retention, and overall quality of the printed materials depend largely on them [17].

Locust bean gum (LBG) is a galactomannan naturally occurring in the endosperm of some *Leguminosae*, mainly extracted from carob tree seeds. It is composed of a linear mannose (M) backbone bearing side chains with a single galactose (G) unit. Galactomannans by themselves can form viscous solutions; however, they are also able to synergistically interact with other biopolymers. The synergistic effects between κ-carrageenan–galactomannan systems depend on the mannose/galactose ratio of the galactomannan backbone [3]. In these mixed systems, the mannose-free regions of the galactomannans are able to associate with carrageenan helices, leading to the formation of elastic and strong gels, offering the potential of improving food products and reducing production costs [18].

In this paper, a very simple and eco-friendly extraction method—water-based—was explored to develop 3D-printed biopolymer-based hydrogels. Three different red seaweed species (*Chondrus crispus*, *Gelidium Corneum*, and *Gracilaria gracilis*) were chosen for its native phycocolloids (carrageenan and agar). The aim of this work was as follows: first, to determine if the green extraction method selected was adequate to obtain bioinks, which form a gel during the printing process; and, second, to establish the best formulation and processing conditions (i.e., optimal thickening agent content and printing temperature) to improve the structure of the printed hydrogel. This evaluation is carried out by analyzing the rheological behavior of the biopolymer solutions in terms of mechanical properties and flow. The influence of the parameters on the gel structure was confirmed by scanning electron microscope (SEM) analysis.

## 2. Results and Discussion

### 2.1. Temperature and Time Sweep: Gels Rheological Behavior during Thermal Cycle

**Figure 1:**
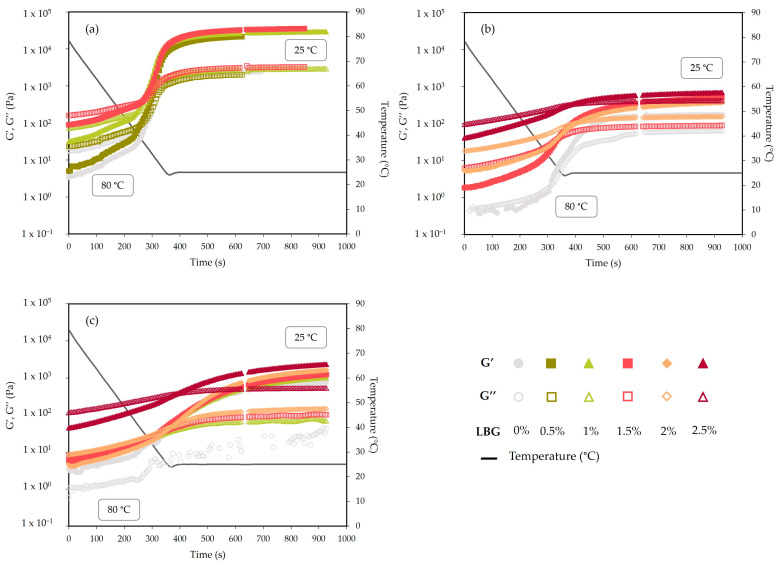
Temperature and time sweeps of *C. crispus* (**a**), *G. gracilis* (**b**), and *G. corneum* (**c**).

The effect of the distinct composition of each seaweed extract, with and without LBG additions, on the gelation mechanism of the phycocolloids-based extracts was rheologically characterized, and the results are presented in Figure 1. As observed, the samples generally showed a similar behavior for viscoelastic moduli as a function of cooling. At the beginning of the cooling cycle from 80 °C, the extracts were fluids with a predominantly viscous character (G″ > G′). Upon further decrease in temperature, the moduli values increased and a cross-over could be detected (i.e., G″ = G′), indicating the beginning of gel formation. This sol–gel transition was rheologically analyzed and is reported in Table 1. The crossover time was termed, in this work, as gelation time (t_gel_) and crossover temperature as gelation temperature (T_gel_). From that moment on, the elastic behavior was predominant (G′ > G″), and eventually, plateau values were achieved for both viscoelastic moduli.

**Figure 2:**
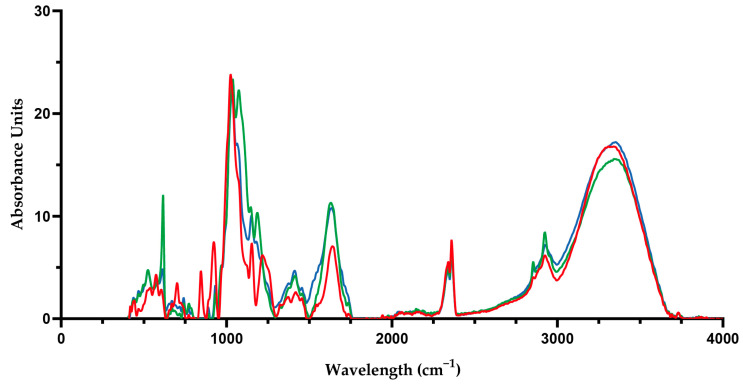
FTIR spectra of seaweed gels: *C. crispus* (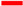), *G. gracilis* (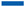), and *G. corneum* (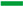).

Concerning the effect of LBG, for *C. crispus* gels, gelation time tended to decrease with increasing additions of LBG (0–1.5% *w*/*w*), whereas gelation temperature seemed to increase. However, no significant influence was detected, so no major effect of the addition of LBG on the gelling behavior of *C. crispus* extracts could be implied. Despite this, the moduli significantly increased, as they increased almost six times their original values when LBG content was 1.5% *w*/*w*. Thus, the addition of LBG promoted an enhancement of their gel strength, influencing the gelation mechanism, which suggests that LBG is playing a significant role in the gel formation process. All *C. crispus* printed gels presented a good shape definition and a self-supporting structure yet maintaining a soft and flexible consistency. Previous studies have shown that LBG additions to κ-carrageenan gels lead to gels with enhanced texture properties [19]. This synergetic behavior between κ-carrageenan and LBG has been extensively reported in the literature [5,20,21,22]. Thus, their good miscibility has been associated with the intermolecular interactions formed between both polysaccharides, which, for example, has led to the formation of films with adequate water vapor barrier and mechanical properties [5]. More specifically, an interaction between the double-stranded helix of κ-carrageenan and the unbranched “smooth” segments of the d-mannose backbone of the galactomannan molecules has been proposed to explain the synergistic mechanism [23]. Previous studies have suggested that in mixed systems containing κ-carrageenan or agar, the gelling mechanism is primarily influenced by these phycocolloids, with other compounds functioning as active particle fillers within the gel network [24,25]. Consequently, analyzing the relevant phycocolloids extracted from seaweed biomass can provide valuable insights into the rheological behavior of the gels. A previous study reports for *C. crispus* (from the same biomass producer as present study—IMTA-cultivated *C. crispus*), a carrageenan yield (aqueous extraction) of 30.6–36.3%. This study also found that the native state of *C. crispus* is a source of copolymers of carrageenan κ/ι [26]. Several studies have shown that FTIR spectroscopy can be used to analyze algae-driven polysaccharides [27,28]. FTIR results (Figure 2 revealed typical bands associated with the presence of copolymers of carrageenan κ/ι. These results showed strong bands associated with κ-carrageenan at 845 cm^−1^, which is assigned to D-galactose-4-sulphate (G4S), and at 930 cm^−1^, indicative of the presence of 3,6-anhydro-D-galactose (DA). Moreover, a lower absorbance at 805 cm^−1^ (3,6-anhydro-D-galactose-2-sulphate) was also exhibited, indicating the presence of sulphate ester in the 2-position of the anhydro-D-galactose residues (DA2S), a characteristic band of ι-carrageenan [29]. Pereira et al. (2009) found similar bands for *C. crispus* collected from the central zone of the western coast of Portugal [27]. Moreover, the typical band (830–820 cm^−1^) for λ-carrageenan was not present in the *C. crispus* gel. The presence of the κ/ι hybrid copolymers contributes to explaining how the obtained *C. crispus* extracts could form stable yet soft gels. While pure κ-carrageenan tends to form hard and brittle gels, in the presence of ι-carrageenan, soft and elastic gels take over [30].

*G. corneum* and *G. gracilis* are the largest sources of agar worldwide, and the crossover between G″ and G′ can be attributed to the formation of a network by aggregation of the agarose helices into larger bundles [31]. No crossover point (G″ = G′) was detected for the *G. corneum* extract without LBG addition, as it already presented a solid-like behavior (G′ > G″ or tan δ < 1), even at 80 °C. In any case, similarly to *C. crispus* extracts, both G′ and G″ increased during the remaining cooling stage from 80 to 25 °C, which can be attributed to additional molecular interactions, leading to the formation of three-dimensional networks [32]. However, the moduli (G′ and G″) after gelation (i.e., at 25 °C) always presented lower values, independently of the LBG content, when compared to *C. crispus* gels. It has been demonstrated that in extraction methods where no pre-treatments (e.g., alkaline solutions) are applied, as is the case in our work, less purified agar-based extracts are produced. These agar-based extracts were reported to form softer gels [31]. Some authors have found that extracts resulting from hot-water extraction processes include other components that, even if they did not have an important effect on the nature of the interactions formed between the agarose chains, did lead to fewer or smaller agarose aggregates, which are responsible for holding the hydrogel network structure. This has been associated with the lower agar (and agarose) content found in hot-water extraction when compared to extraction processes where strong alkali are employed [33].

Conversely to *C. crispus* gels, the addition of LBG led to crossover points (G″ = G′) to happen later (i.e., at a lower temperature) in the cooling step. A similar behavior was reported by Sousa and Gonçalves (2015), where agar/LBG gels presented lower gelation temperatures and higher gelation times, compared to pure agar gels [25]. Previous studies reported gelling temperatures for *G. gracilis* and *G. corneum* biomasses (from the same producers as the present study—Algaplus and Iberagar) of 42.2 °C and 34.4 °C, respectively [34,35]. In our study, gelling temperatures from pure *G. gracilis* and *G. gelidium* extracts (i.e., without LBG) presented lower T_gel_ compared to the previous reported values. These findings could indicate an influence of other compounds present in the extract (e.g., proteins, lipids, ash, starch), and/or their interactions with agar, on the formation of the gels [36].

As mentioned before, no cross-over point (G″ = G′) was detected for the *G. corneum* extract without LBG addition, as that extract already displayed a predominantly elastic behavior at 80 °C. However, an increase in G′ with cooling reveals a gel structure reinforcement. Sousa and Gonçalves (2015) reported no cross-point for a mixed system of agar/LBG (25–75 ratio, respectively). However, at a 50–50 ratio, a crossover of the moduli was reported [25]. In our study, although increasing additions of LBG did not hinder the occurrence of the crossover between G′ and G″, the gelling properties of *G. gracilis* and *G. gelidium* gels seem to be greatly influenced by increasing additions of LBG. Particularly, *G. gelidium* finds a reversal from solid-like behavior to fluid-like behavior at 80 °C due to the addition of LBG. This should be related to the reported less coarse network found when LBG was added to agar solutions, leading to a decrease in the gelation point and a reduction in elastic moduli [26]. The reversal from solid-like to fluid-like behavior at 80 °C might be a good point for 3D printing, as those extracts might flow in an easier way through the nozzle and then gel once they are deposited on the printing bed at lower temperatures. This, together with the higher viscoelastic moduli due to the presence of the thickening agent, can contribute to a better definition for printed samples containing LBG.

Immediately after the temperature sweep, the gel maturation kinetics of the extracts were monitored through the evolution with time of G′ and G″ moduli during an isothermal stage at 25 °C (Figure 1). While *C. crispus* gels reached the steady state revealing constant values of both moduli, *G. gracilis* and *G. corneum* gels, particularly the latter, showed a slower equilibration step. The maturation pattern observed in the gels of *G. gracilis* and *G. corneum* follows the typical behavior of biopolymer gelation. Initially, G′ shows a rapid increase, followed by a slower progression. This is generally attributed to the ongoing reorganization of polymeric molecules within the gel network. [37].

Table 2 reports the viscoelastic properties (G′, G″, and tan δ) for the seaweed gels, at 80 °C (before gelation) and 25 °C (after gelation). Overall, increasing additions of LBG led to a decrease in elasticity (tan δ increase) of all seaweed gels. Specifically, the gels of *G. gracilis* displayed a significant increase of 244% in tan δ when transitioning from 0% LBG to 2.5% LBG gels. Sousa and Gonçalves (2015) also reported significant elasticity losses (tan δ increase) in agar/LBG binary systems [25]. In contrast, all *C. crispus* gels presented a comparable elastic response (tan δ circa 0.07–0.1). The same behavior was observed for *G. corneum* gels, up to 2% LBG addition.

For all seaweed extracts, G′ at 25 °C is significantly higher than at 80 °C, as expected after gelation. At 25 °C, *C. crispus* gels exhibited a decrease in G′ values (G′ _25 °C_), when 0.5% LBG was added to the gel. However, further additions led to an increase in the elastic modulus, reaching a similar G′ value of the gel without LBG. This could be explained by the interference effect of the low content of LBG on the self-association of κ-carrageenan chains [5]. Further LBG additions seem to have reinforced the gel’s network structure (increased G′ values). A similar structuring effect can be observed for *G. corneum* and *G. gracilis* gels. Overall, LBG additions contributed to elevate gel strength of these seaweeds. The exception is for the *G. gracilis* gel at 2% LBG addition, where G′ exhibits a sudden decrease. A previous study also reported a synergy for agar extracted from a *Gelidium* sp. and LBG in 1:9 proportion, when a maximum hardness of the gel was achieved. When this ratio changed to 1:4, the gel returned to exhibit the gel strength of agar, i.e., the synergistic behavior was lost [38]. The same authors stated that such agar-LBG synergism is not possible for agars extracted from *Gracilaria* spp. Findings from another study confirm a lack of agar/LBG synergy for *Gracilaria vermiculophylla*. This study reported a gradual decrease in gel rigidity (G′ decrease) with LBG addition in mixed systems of agar/LBG, with higher concentrations of LBG [26]. In a more detailed analysis, Khoobbakht et al. (2024) observed that agar/LBG (commercial hydrocolloids) formed harder gels when mixed in a 4:1 ratio compared to a 1:1 ratio. [39]. Thus, while previous studies suggest a limited synergy between agar and LBG, our study presents a contrasting perspective. Our findings indicate an enhancement of the viscoelastic properties of gels derived from *G. corneum* and *G. gracilis* when LBG is added.

**Figure 3:**
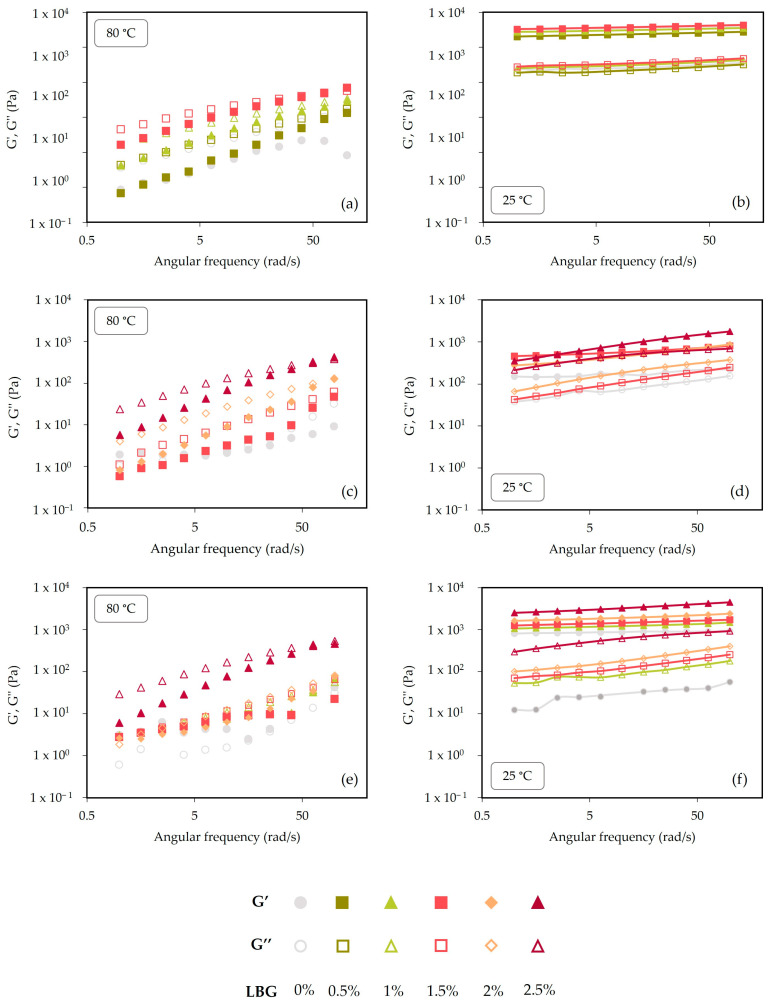
Frequency sweeps of *C. crispus* (**a**,**b**), *G. gracilis* (**c**,**d**), and *G. corneum* (**e**,**f**), at 80 and 25 °C, respectively.

Contrarily to the viscoelastic properties at the crossover point (Table 1), at 25 °C (Table 2), *G. corneum* gels exhibit higher viscoelastic properties (G′, G″) than *G. gracilis*. These findings reflect the gel strength of *G. corneum*, accordingly shown on the mechanical spectra (Figure 3). These results can generally be attributed to (i) the yield of extracted agar and to (ii) the agar quality of each of the seaweeds. In the context of agar yield, previous studies report considerable variability of agar yields, extracted from *G. corneum* (from the same producer as in our study). A previous study on the influence of seasonal harvesting on the biochemical characteristics of *G. corneum* revealed that its agar aqueous extraction yield ranged between 5.99% (spring) and 8.70% DW (winter) [40]. Martínez-Sans et al. (2021) reported a yield of 10–12% for *G. corneum* (as *G. sesquipedale*), while Gomes-Dias et al. (2022) obtained an extraction yield of 8.4% for native agar using a conventional hot-water bath extraction method (95 °C, 90 min) [35,41]. On the other hand, other studies have reported higher yields for agar extracted from *G. corneum* through aqueous extraction methods, ranging from 21% to 48% [42,43]. Native agar yields from *Gracilaria* typically range around 10–15% but can increase when using alkali pretreatment (15–33%) [44]. A previous study reported an agar yield of 14% for a similar *Gracilaria gracilis* biomass, obtained from the same producer as the present study [34]. Regarding the agar quality, *Gelidium* spp. is known for presenting a better agar quality than *Gracilaria* spp. This is due to the natural internal desulfation of *Gelidium*, modulated by enzymatic processes. On the other hand, *Gracilaria* presents polysaccharides which are typically more sulfated, and the conversion of these sulfated groups (i.e., L-galactose-6-sulphate to 3,6-anhydro-L-galactose) does not occur in sufficient quantity during the seaweed’s lifespan [38]. As a result, the *Gracilaria* species typically requires alkali pre-treatments to improve the gelling capacity of its agar gels [45,46]. In summary, the findings suggest that native agars from *G. corneum* exhibit superior gelling properties compared to *G. gracilis*, explaining the significantly higher values of G′ at 25 °C for the former (Table 2). Nonetheless, it is noteworthy to note that despite the increase in G′ _25 °C_ values (over G′ _80 °C_), the values obtained for *G. cornem* and *G. gracilis* gels without LBG addition (737.8 and 446.9 Pa, respectively) are substantially lower than values reported in the literature. Gomes-Dias et al. (2024) reports 8838 Pa for G′ at 20 °C, for hot-water extracted agars (95 °C, 180 min) from *G. corneum*. The same study reports a contrastingly high G′ value (2567 Pa) at 20 °C for agar extracted from *Gracilaria vermiculophyla* [47]. One possible explanation for the lower values obtained in our study can be the purification step for agar that most studies perform when analyzing gelling properties of native seaweed-derived hydrocolloids. The lack of the purification step in the present study could have led to the presence of other components in the seaweed extracts, such as proteins, which have shown to give rise to the formation of softer hydrogels [31]. Nevertheless, non-purification is a deliberate strategy in this study, considering the positioning of the gels within the context of clean label foods. Moreover, even when comparing the extraction method employed in this study with similar extraction methods (hot-water extractions), it appears that most studies have used longer extraction times (>180 min) and higher temperatures (>95 °C) than ours (70 min, 90 °C).

### 2.2. Frequency Sweeps: Gels Characteristics

Frequency sweeps, shown in Figure 3, were performed both in the extract solutions at 80 °C and in the gels formed after cooling to 25 °C, and demonstrate the distinct behavior of the seaweed extracts at the two physical states for different LBG contents. At 80 °C (Figure 3a,c,e), seaweed extracts were presented in a liquid-like state. At this temperature, G′ and G” values were similar for all the seaweed extracts and increased with frequency. G″ was always above G′, which is a behavior typical of entangled solutions [31]. While *C. crispus* extracts show an increase in elastic properties, manifested by a notable rise in G′ and a reduction in tan δ (Table 2), as the content of LBG content increases; for *G. gracilis* and *G. corneum*, tan δ is always higher for the systems containing LBG, which would indicate a growing importance of their viscous behavior, which could be negative for attaining self-supporting gels in the 3D-printing process.

At 25 °C (Figure 3b,d,e), seaweed extracts acquire a solid-like texture and G′ is always above G″. At 25 °C, *C. crispus* extracts showed a behavior typical of strong gels, where G′ was at least one order of magnitude higher than G″, and both moduli showed little frequency dependence within the studied range (0.1–100 rad/s). This behavior should be beneficial for the 3D-printing process, as the spread of the sample on the printing bed would be hindered. The same could not be observed for *G. gracilis* and *G. corneum* gels. Although G′ was always above G″, both these gels presented a greater frequency dependence, indicating a weak-gel-like behavior. Nonetheless, *G. corneum* gels presented enhanced gel strength (higher G′ values) than *G. gracilis* gels (Table 2). Increasing additions of LBG to gels seemed to increase the slope of G′ and G″ along the frequency range (Figure 3). Sousa and Gonçalves (2015) reported an increase in frequency dependence of the moduli at higher LBG concentrations of agar/LBG binary systems [25].

The results obtained from the mechanical spectra (Figure 3) can be further explained by the composition of seaweeds used in this study, as the polysaccharides’ composition has a direct effect on the gelling mechanism and gel strength. According to Gomes-Dias et al. (2024), *Gelidium corneum* biomass (cultivated and harvested by the same manufacturer from the present study—Iberagar) presents galactans (37%) and glucans (14%) in its sugar composition, although 28% of these polysaccharides are of water extractives [47]. Silva-Brito et al. (2021) studied the sugar composition of *G. gracilis* biomass (from the same producer as our biomass—Algaplus) and reported 24.4% of galactans, 3.5% of glucans, and 2.6% of arabians [35]. Moreover, Nova et al. (2023) reported for the same *G. gracilis* biomass (cultivated and harvested by the same producer), the presence of galactose as the main sugar residue (34 mol%), followed by 3,6-anhydrogalactose (25 mol%) and glucose (19 mol%) [48]. The presence of glucose is likely due to the presence of floridean starch, a storage polysaccharide [36].

The degree of sulfation and the content on 3,6-anhydro-L-galactose (3,6-AG) is also revealed to be an important factor to consider when evaluating the quality of agars [36]. The conversion of L-galactose-6-sulphate to 3,6-anhydro-L-galactose is associated with an increase in gel strength, as well as in gelling temperatures [49]. Consequently, the lower the sulphates/3,6-AG ratio, the higher the gelling properties [47]. Gomes-Dias et al. (2024) reported a sulfate content of 0.49% and a 3,6-AG content of 28.2% for agar extracted from *G. corneum* (hot-water extraction). The same study reports a sulfate content of 2.1% and a 3,6-AG content of 25.3% for agar extracted from *Gracilaria vermiculophyla* [47]. For the same seaweed, other studies have reported higher values for sulfate and 3.6- AG content. Sousa et al. (2010) reported a 3,6-AG content of 31.3% and a sulfate content of 1.78% for agar extracted from *G. vermiculophylla,* produced in an IMTA system and subjected to a traditional extraction method (alkali treatment and acid neutralization) [49]. For the same red seaweed (without alkali pre-treatments), Villanueva et al. (2009) reported a content of 3,6-AG of ~32–33% and a sulfate content of 2.2–2.4% [50]. Nonetheless, for agar extracted from *G. gracilis* (harvested from the southern Atlantic coast of Morocco) Belattmania et al. (2021) reported a 3,6-AG content of 5.7% and a sulfate content of 0.7% [51].

Previous studies have found a negative correlation between sulfate content and gel strength and gelling temperature, while 3,6-AG content has been found to have the opposite correlation with these gelation properties [49,50]. In the present study, all seaweed biomasses underwent a water-based extraction without alkaline pre-treatments. Consequently, the sulfate content was anticipated to be higher, while the 3,6-AG content was expected to be lower, in comparison to prior studies employing traditional or alternative extraction methods. Gomes-Dias et al. (2022) observed that conventionally extracted agar presented double the sulphate content of the autohydrolysis extracted agar, which led to lower viscoelastic moduli values [35]. Additionally, as previously mentioned, it is noteworthy that agars from *Gelidium* spp. typically present a low degree of substitution and, thus low sulfate content, resulting in agars with high gel strength, compared to *Gracilaria* spp. [52]. FTIR results (Figure 2) revealed that the band at 930 cm^−1^ in the spectra, corresponding to 3,6-anhydro-galactose, was more intense in the *G. gracilis* gels than in the *G. corneum.* However, a more intense broad band for *G. gracilis* in the 1240–1260 cm^−1^ spectral region, associated with the vibration of the sulphated groups, contributes to a higher sulphates/3,6-AG ratio. As sulfate groups are related to a lower gel strength of agar, agar in *G. gracilis* is considered to present lower agar quality, due to the higher presence of α-l-galactose 6-sulphate units detected in FTIR [53]. Sulphur content in the seaweed extracts can not only be attributed to the sulphate substitution in the agar component, but also to the presence of other sulphated components such as proteins [8]. *G. gracilis* biomass is reported to have a higher protein content (21%) than *G. corneum* biomass (10.4–19.4%). 

This divergence in seaweed composition contributes to elucidating the heightened gelling properties observed in *G. corneum*, evidenced by increased gel strength (higher G′ values) and a higher gelling temperature (T_gel_) when lower LBG additions (<1% *w*/*w*) are applied (Table 1).

### 2.3. Flow Curves: Gel Viscosities

**Figure 4:**
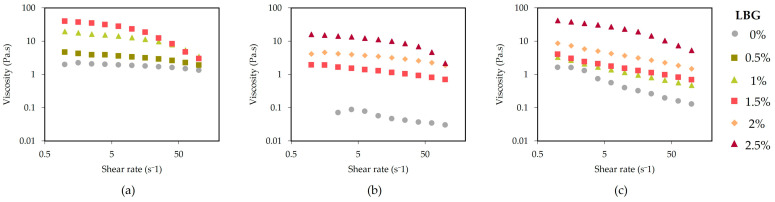
Flow curves of *C. crispus* (**a**), *G. gracilis* (**b**), and *G. corneum* (**c**), at 80 °C (1–100 s^−1^).

Figure 4 presents the steady shear flow behavior of seaweed extracts at 80 °C, over the selected range of shear rates (1–100 s^−1^). Results obtained from viscosity curves showed a shear-thinning behavior, with higher viscosities at low shear rates and lower viscosities at high shear rates. Such flow behavior is necessary for a successful 3D extrusion. During printing, high shear rates are generated in the nozzle walls, and extrusion is thus facilitated if viscosity decreases. The process is reversed when a food material is layered on the printing platform, and viscosity increases due to the reduction in shear rates, contributing to forming a self-supporting material [54].

Table 3 reports the viscosity values obtained at a selected shear rate (100 s^−1^) for all seaweed extracts. This shear rate value was selected as it is the closest shear rate at the nozzle, based on estimated values obtained from the Weissenberg–Rabinowitsch–Mooney equation.

Results show that flow properties were dependent on LBG concentration: higher viscosity with higher additions of LBG, demonstrating the synergetic effect: η algae_extract_ + LBG > η algae_extract_. Similarly to viscoelastic properties, *C. crispus* gels obtained the highest values, followed by *G. corneum* and *G. gracilis*.

Hernández et al. (2001) reported increased viscosity for a binary system composed of κ-carrageenan and λ-carrageenan with LBG (LBG + κ and LBG + λ), compared to single systems of any of the studied gums. These findings align with the results obtained in this study, reinforcing the presence of synergistic effects—specifically, an increase in viscosity—resulting from the combination of LBG with carrageenan [55].

As can be clearly observed in Supplementary Table S1, where shift factors for viscosity and shear rate were used to overlap every seaweed extract system to the corresponding system without LBG addition (LBG 0%) (master curve for every seaweed extract in Supplementary Figure S1), *G. corneum* is the seaweed most affected by the addition of LBG, as at 1.5% LBG, the viscosity enhancement was almost 4 or 5 times that found for *C. crispus* or *G. gracilis*, respectively.

### 2.4. Printed Gels

**Figure 5:**
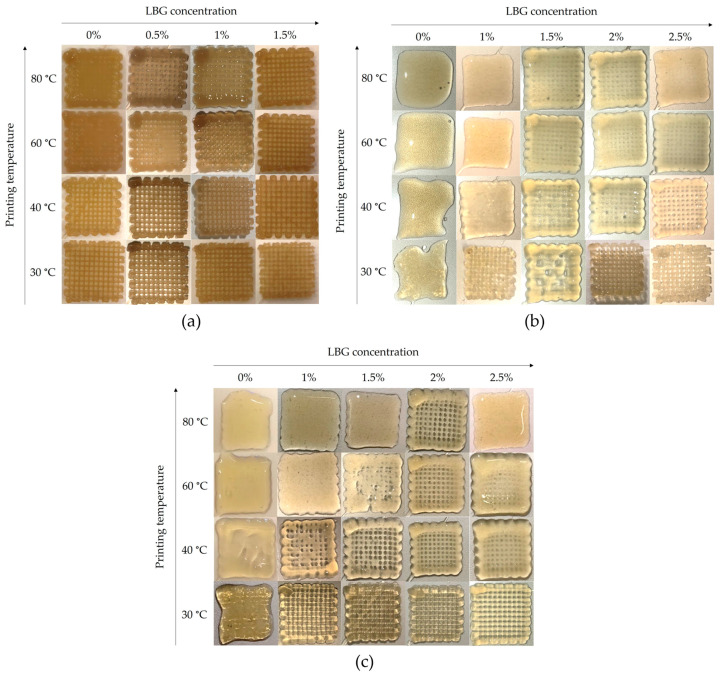
Visual effect of LBG additions (0–2.5% *w*/*w*) and printing temperature (80–30 °C) on gel’s structure of *C. crispus* (**a**), *G. gracilis* (**b**), and *G. corneum* (**c**).

In Figure 5, the visual representation of printed gels from *C. crispus* (a), *G. gracilis* (b), and *G. corneum* (c) is shown. The figures illustrate the impact of cooling the printing temperature (from 80 to 30 °C) and increasing LBG content on the appearance of the seaweed gels.

*C. crispus* exhibited the best gel structure, confirmed by its viscoelastic properties (G′, G″ and tan δ) (Table 1 and Table 2). Moreover, all *C. crispus* printed gels (Figure 5a) presented a good shape definition and a self-supporting structure yet maintaining a soft and flexible consistency, which should be related to the abovementioned rheological properties, and to the fact that texture properties are affected by LBG additions to κ-carrageenan gels. During deposition of layers and cooling of the *C. crispus* extracts, a typical gel structure was obtained, and even for extracts without LBG addition, a well-defined 3D structure was produced. Contrastingly, printing definition was very poor for *G. corneum* and *G. gracilis* gels without LBG, as the material flowed when deposited (Figure 5b,c). With increasing LBG additions, both *G. corneum* and *G. gracilis* acquired an improved shape definition and increased gel strength (Table 1 and Table 2). Nevertheless, it was observed that additional additions of LBG had a more pronounced impact on the gel structure of *G. gelidium* (Figure 5c) in comparison to *G. gracilis* gel (Figure 5b). This should be related to *G. corneum* gels exhibiting higher viscoelastic properties (G′, G″) than *G. gracilis*, which reflect the gel strength of *G. corneum*, accordingly shown on the mechanical spectra (Figure 3) and visibly noticeable in Figure 5c, where *G. corneum* gels acquire a better-defined shape with increasing additions of LBG. Moreover, a better shape definition of *G. corneum* gels can be further supported by the gels’ higher values of viscosity, as evidenced in Table 3. In contrast, a reduction in the printing temperature seemed to produce a greater improvement of shape definition in *G. gracilis* gel, when compared to *G. gelidium* gel. Lower printing temperatures increase viscosity and may reduce the spreading effect of the filaments during deposition.

**Figure 6:**
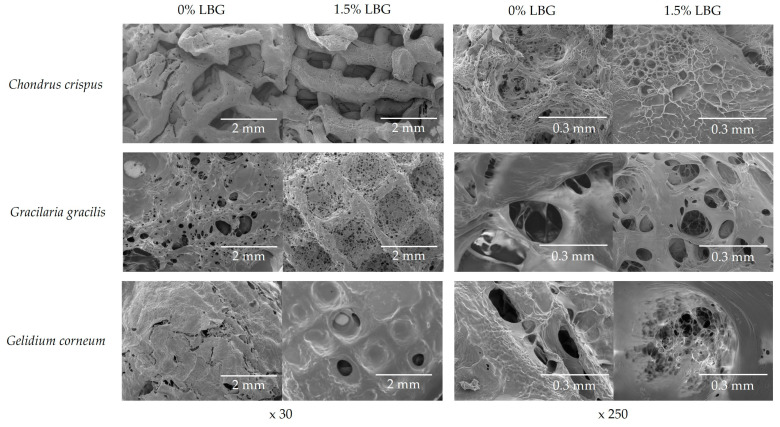
SEM images of *C. crispus*, *G. gracilis*, and *G. Corneum* 3D gels, with and without LBG addition, under 30× and 250× magnifications.

SEM was used to characterize the microstructure of the gels. Figure 6 shows the internal structures of the *C. crispus*, *G. gracilis*, and *G. corneum* gels, printed at 25 °C, with (1.5%) and without LBG addition. Under a 30× magnification, it was possible to observe the printed layer filaments of the gels at 1.5% LBG addition while, without LBG, the printed filaments could not be observed, except in the *C. crispus* gel. For this seaweed, the impact of LBG addition to the structure was lower, as can be observed in Figure 5a. These results agree with the mechanical spectra obtained from frequency sweeps (Figure 3), which show little frequency dependence, indicating the presence of a strong gel.

Under a 250× magnification, the morphology of the gel and the size and number of pores illustrate the molecular interactions established with and without LBG addition to seaweed gels. Overall, 1.5% LBG addition resulted in denser gels characterized by smaller-sized pores. This effect was particularly noticeable in *C. crispus* and *G. gelidium* gels, confirming the stronger synergistic interaction among the gelling components (κ/ι-carrageenan and agar, respectively) in these gels. In contrast, *G. gracilis* exhibited larger pores and a less dense microstructure. All the seaweed gels presented a heterogeneous structure. Previous studies have shown that gels of mixed systems generally present a more heterogenous structure than gels of individual gelling components [56].

## 3. Conclusions

The present study investigated the gel-forming kinetics and gel properties of algal polysaccharides obtained in a green extraction process from three red seaweed biomasses (*Chondrus crispus*, *Gracilaria gracilis*, and *Gelidium corneum*). Locust bean gum (LBG) was added to the final extracts, and gelation was monitored in situ. Results showed a similar gelation behavior for all seaweed extracts during the cooling cycle, transitioning from a liquid-like state to a final gel formation, at the crosspoint between G′ and G″. *C. crispus* gels exhibited increased gel strength (higher G′) and more stable gels (shorter maturation time to obtain steady gel structures). This structuring effect was additionally observed on the microstructure of the gels. *G. corneum* and *G. gracilis* exhibited lower viscoelastic properties, yet their gel strength noticeably improved with the addition of LBG, indicating a synergistic interaction between the seaweed extracts and LBG. This synergy proved to be necessary to obtain self-supporting structures from *G. gracilis* and *G. corneum* extracts, during the printing process. Similarly, viscosity of gels was likewise enhanced by the increasing additions to the seaweed extracts. Nevertheless, flow curves proved that gels presented a suitable flow behavior for extrusion: a shear-thinning behavior with increasing shear-rates. Additionally, printing at lower temperatures seemed to have improved the shape definition of the printed gels.

These findings confirm that a green-extraction can be applied to develop algal-based extracts with gelling capacity. Additionally, a synergetic interaction between extracts and LBG enhanced the gels’ suitability for printing. This could be used in several applications, such as the design of novel food products. The effect of different thickening agents on the features of the bioinks and the resulting 3D-printed gels should be considered in future research.

## 4. Materials and Methods

### 4.1. Materials and Sample Preparation

Materials. Three different species of red algae (Rhodophyta) were selected for the preparation of the biopolymer solutions: *Chondrus crispus*, *Gelidium Corneum*, and *Gracilaria gracilis*. *Chondrus crispus* is typically rich in carrageenan (being κ-carrageenan and ι-carrageenan, able to form gels), while agar, a gelling, thickening, and stabilizing agent, is more abundant in *Gelidium Corneum* and *Gracilaria gracilis*. *C. crispus* and *G. gracilis* were purchased from AlgaPlus (Ilhavo, Portugal) and *G. corneum* from Iberagar-Sociedade Luso-Espanhola de Colóides Marinhos S.A. (Coina, Portugal). According to the datasheet of the producer, *C. crispus* and *G. gracilaria* were cultivated in an integrated multi-trophic aquaculture system (Aveiro, Portugal). The seaweeds were washed with seawater and dried in an air-tunnel set at 30 °C, until the desired moisture content (12%) was reached, followed by milling and sieving (<0.25 mm). Regarding *G. corneum*, according to the manufacturer, the algae undergo a drying process in the sun, are milled, and finally sieved (3 mm).

The proximate composition for *C. crispus*, provided by the manufacturer, is as follows: 12% moisture, 1% lipids, 17.7% carbohydrates (<0.01% sugars), 17.3% protein, and 38.1% dietary fiber. For *G. gracilaria*, it is as follows: 12% moisture, 1.2% lipids, 8.2% carbohydrates (0.2% sugars), 21% protein, and 38.8% dietary fiber. For *G. corneum*, a previous study using a similar biomass from the same manufacturer obtained the following: 9.2–19.8% ash, 0.7–4.7% lipids, 24.8–39.5% carbohydrates, and 10.4–19.4% crude protein [43,44,57]. These red algae were selected for their native phycocolloids and gelling ability, crucial for the 3D-printing process.

Sample preparation. Seaweed solutions were prepared following a 2-step alternative extraction method. First, seaweeds (in dry powder) were hydrated in water (1:10 *w*/*w*, seaweed/water), for 30 min, at room temperature. Then, the seaweed solutions were heated to 90 °C (70 min) with constant stirring. Finally, the solutions were centrifuged (40 °C, 10,000 rpm, 15 min), and the supernatants were collected to be printed. Once printed, gels from the different species of seaweeds did not all present the same definition of shape. To improve the shape quality of the printed gels, the addition of locust bean gum (LGB) to the supernatants was studied, as LBG presents a synergy with naturally present phycocolloids in Rhodophyta (carrageenan and agar). LBG was added to supernatants immediately following centrifugation, under strong stirring to ensure full dissolution. Based on preliminary tests, LBG additions to supernatants were set as follows: *Chondrus crispus* (0–1.5% *w*/*w*), *Gelidium Corneum* (0–2.5% *w*/*w*), and *Gracilaria gracilis* (0–2.5% *w*/*w*).

### 4.2. Three-Dimensional-Printing Process

The printing process was performed using the Genesis™ 3D printer (3D Biotechnology Solutions, Brazil), following the LBG addition to the supernatants.

Printing of the gels took place at a different range of temperatures (80–30 °C) to study the influence of printing temperature on the quality of shape of the printed gels. To do this, syringes (22 G nozzle) were loaded with the seaweed extracts and were pre-heated beforehand according to the selected printing temperature, while the printing platform was always kept at room temperature. After 15 min of equilibration time in the syringe, the extracts were printed at 40 mm/s, forming a total of 8 layers of a cube design (20 × 20 × 20 mm).

### 4.3. Rheology Characterization

Rheology characterization tests were performed to study the viscoelastic properties of the algae-based biopolymers solutions, in terms of storage (G′) and loss (G″) moduli, using a strain-controlled rheometer (Discovery HR 10, TA instruments, New Castle, DE, USA), equipped with a Peltier plate temperature system. A serrated parallel plates geometry (PP20) was used, with a gap of 1 mm.

Before starting measurements, hot biopolymer solutions were directly placed on the preheated parallel plates geometry and coated with paraffin oil to avoid evaporation.

Determining LVE. Firstly, the linear viscoelastic region (LVE) was determined via amplitude sweep tests, carried out at a fixed frequency (6.28 rad/s), at 80 °C. All measurements were performed within the LVE, as at this range, all dynamic rheological parameters are independent of applied shear strain.

First frequency sweep. Secondly, frequency sweep tests were carried out within a frequency range varying from 1 to 100 rad/s (ω), at a fixed temperature (80 °C), to study the viscoelastic properties.

Temperature sweep. Following frequency sweeps, temperature sweeps were run, applying a cooling temperature ramp from 80 to 25 °C (ω = 6.28 rad/s, 10 °C/min). During temperature sweeps, the transition from sol-gel was monitored, which corresponds to tan δ = G″/G′ = 1. The temperature and time at which the crossover between G′ and G″ occurred, i.e., G″ > G′, was noted as an empirical indication of gelling temperature (T_gel_) and time (t_gel_), respectively [58].

Time sweep. Time sweeps were performed (30 min, ω = 6.28 rad/s, 25 °C) to obtain the gel maturation kinetics.

Final frequency sweep. A final frequency sweep was performed on the gels, from 1 to 100 rad/s (ω), at a fixed temperature (25 °C), to analyze the influence of the temperature ramp on the viscoelastic properties.

Flow curves. Flow sweeps were performed (80 °C) to study the viscosity (η) of the biopolymer solutions as a function of a selected range of shear rates (1–100 s^−1^).

### 4.4. Fourier Transform Infrared (FTIR) Spectroscopy

Seaweed extracts at 80 °C were cast in Petri dishes and dried overnight. FTIR spectra of films formed from seaweed extracts were performed with an ATR objective in an Invenio X spectrometer (BRUKER, Billerica, MA, USA). Spectra were collected in the wavelength between 4000 and 400 cm^−1^, with a resolution of 4 cm^−1^.

### 4.5. Scanning Electron Microscope (SEM)

The microstructures of the gels were investigated via scanning electron microscopy (SEM) using a tabletop scanning electron microscope (SEM) (TM3030 PLUS, Hitachi, Japan). Samples were printed, and immediately, small cuts were performed on their surface using a scalpel to promote better observation of the inner microstructures. The samples were placed on the specimen holder, at −4 °C, in a vacuum chamber. Observations were performed under different magnifications (30× and 250×).

### 4.6. Statistical Analysis

Analysis of variance (ANOVA) was performed on Minitab (version 17, Pennsylvania, USA) to evaluate significant (*p* < 0.05) differences between samples. In case of significant differences, multiple pairwise comparisons were performed with Tukey’s Honest Significant Difference test (HSD) at 95% confidence level.

## Figures and Tables

**Table 1:** Viscoelastic properties of seaweed (*C. crispus*, *G. gracilis,* and *G. corneum*) extracts, with different LBG concentrations, at the crosspoint G′ = G″: gelation time (t_gel_), gelation temperature, (T_gel_), and gelation modulus (G_gel_).

Seaweed Extract	LBG (%)	t_gel_ (s)	T_gel_ (°C)	G′_gel_ (Pa)
*C. crispus*	0.0	257.39 ± 23.88 ^a^	35.45 ± 3.99 ^a^	64.17 ± 19.36 ^c^
	0.5	244.15 ± 12.05 ^a^	37.19 ± 2.64 ^a^	85.47 ± 8.65 ^c^
	1.0	229.13 ± 16.10 ^a^	39.31 ± 2.67 ^a^	179.83 ± 8.72 ^b^
	1.5	225.59 ± 46. 39 ^a^	39.90 ± 7.72 ^a^	358.43 ± 41.90 ^a^
*G. gracilis*	0.0	298.92 ± 31.93 ^b^	27.88 ± 5.06 ^a^	2.66 ± 0.50 ^a^
	1.5	318.84 ± 2.11 ^b^	25.73 ± 2.11 ^a^	45.27 ± 10.30 ^b^
	2.0	397.79 ± 0.02 ^a^	24.99 ± 0.02 ^a^	116.90 ± 13.29 ^c^
	2.5	394.61 ± 0.01 ^a^	25.00 ± 0.01 ^a^	325.64 ± 15.52 ^d^
*G. corneum*	0.0	*No crosspoint*		
	1.0	298.54 ± 12.27 ^c^	29.89 ± 2.68 ^a^	23.02 ± 3.19 ^a^
	1.5	328.92 ± 8.17 ^b^	25.36 ± 1.32 ^b^	32.34 ± 5.03 ^b^
	2.0	377.16 ± 3.73 ^a^	25.03 ± 0.01 ^b^	56.74 ± 11.73 ^b^
	2.5	387.76 ± 12.25 ^a^	25.01 ± 0.01 ^b^	422.33 ± 52.79 ^b^

Values are given as mean ± standard deviation (n = 3). Means (for the same sample) with different letters, within the same column, differ significantly (*p* < 0.05).

**Table 2:** Viscoelastic properties of seaweed (*C. crispus*, *G. gracilis,* and *G. corneum*) extracts, with different LBG concentrations, before (80 °C) and after (25 °C) at the crosspoint: elastic modulus at 80 °C (G′ _80 °C_) and at 25 °C (G′ _25 °C_), tan δ at 80 °C (tan δ _80 °C_) and at 25 °C (tan δ _25 °C_).

Seaweed Extract	LBG (%)	G′ _80 °C_ (Pa)	G′ _25 °C_ (Pa)	tan δ _80 °C_	tan δ _25 °C_
*C. crispus*	0.0	3.95 ± 0.76 ^b^	30,746.25 ± 5277.21 ^a^	3.91 ± 1.55 ^a^	0.07 ± 0.00 ^b^
	0.5	5.73 ± 1.03 ^b^	20,580.05 ± 1413.15 ^b^	4.16 ± 0.71 ^a^	0.09 ± 0.00 ^a^
	1.0	36.54 ± 6.65 ^b^	27,675.65 ± 23,10.27 ^a,b^	2.12 ± 0.22 ^b^	0.10 ± 0.01 ^a^
	1.5	129.75 ± 60.01 ^a^	30,137.03 ± 4766.15 ^a^	1.53 ± 0.26 ^b^	0.10 ± 0.01 ^a^
*G. gracilis*	0.0	0.32 ± 1.14 ^b^	446.99 ± 309.49 ^a^	1.03 ± 1.79 ^a^	0.18 ± 0.18 ^b^
	1.5	5.47 ± 5.11 ^b^	579.51 ± 110.26 ^a^	2.60 ± 1.13 ^a^	0.19 ± 0.02 ^b^
	2.0	5.45 ± 0.13 ^b^	429.84 ± 80.71 ^a^	3.56 ± 0.25 ^a^	0.40 ± 0.02 ^a,b^
	2.5	39.14 ± 3.19 ^a^	662.01 ± 44.31 ^a^	2.35 ± 0.02 ^a^	0.62 ± 0.01^a^
*G. corneum*	0.0	3.17 ± 0.42 ^b^	737.84 ± 65.50 ^e^	0.29 ± 0.17 ^d^	0.05 ± 0.01 ^c^
	1.0	5.97 ± 0.31 ^b^	943.61 ± 136.30 ^d^	1.28 ± 0.00 ^c^	0.07 ± 0.00 ^b^
	1.5	6.44 ± 0.50 ^b^	1259.55 ± 30.00 ^c^	1.35 ± 0.06 ^c^	0.09 ± 0.00 ^b^
	2.0	5.88 ± 2.07 ^b^	1718.15 ± 123.68 ^b^	1.78 ± 0.2 ^b^	0.09 ± 0.00 ^b^
	2.5	66.50 ± 30.17 ^a^	2338.98 ± 22.61 ^a^	2.32 ± 0.41 ^a^	0.24 ± 0.00 ^a^

Values are given as mean ± standard deviation (n = 3). Means (for the same sample) with different letters, within the same column, differ significantly (*p* < 0.05).

**Table 3:** Flow properties of seaweed (*C. crispus*, *G. gracilis*, and *G. corneum*) gelled extracts, with different LBG concentrations, at 80 °C: viscosity at (η_100_).

Seaweed Extract	LBG (%)	η_100_ (Pa·s)
*C. crispus*	0.0	1.29 ± 0.05 ^d^
	0.5	2.09 ± 0.19 ^c^
	1.0	3.48 ± 0.04 ^b^
	1.5	2.83 ± 0.15 ^a^
*G. gracilis*	0.0	0.04 ± 0.03 ^c^
	1.5	0.71 ± 0.04 ^b^
	2.0	1.70 ± 0.48 ^a^
	2.5	1.93 ± 0.65 ^a^
*G. corneum*	0.0	0.11 ± 0.02 ^c^
	1.0	0.49 ± 0.02 ^b,c^
	1.5	0.65 ± 0.04 ^b,c^
	2.0	1.37 ± 0.11 ^b^
	2.5	4.32 ± 1.00 ^a^

Values are given as mean ± standard deviation (n = 3). Means (for the same sample) with different letters, within the same column, differ significantly (*p* < 0.05).

## Data Availability

All data and materials are available on request from the corresponding author. The data are not publicly available due to ongoing research using a part of the data.

## Supplementary Materials

The following supporting information can be downloaded at: https://www.mdpi.com/article/10.3390/gels10030166/s1, Figure S1: Master curve for the different LBG contents for every seaweed extract, using every system without LBG as reference; Table S1: Shift factor for viscosity and shear rate to overlap every seaweed extract system to the corresponding system with-out LBG addition (LBG 0%).
