# Supplementary material for: Three-Dimensional Printing of Red Algae Biopolymers: Effect of Locust Bean Gum on Rheology and Processability
Source: Gels. 2024 Feb 23;10(3):166. doi: 10.3390/gels10030166 (PMC10970507; doi:10.3390/gels10030166)
Supplement: Supplementary file 1 [file gels-10-00166-s001.zip › Supplementary Table S1.pdf]

Supplementary Table S1. Shift factor for viscosity and shear rate to overlap every seaweed extract system to the corresponding system without LBG addition (LBG 0%)

Seaweed extract	LBG (%)	viscosity shift factor, $(a_c)_I$	shear rate shift factor, $(a_c)_{II}$
C. crispus	0	1.00	1.00
	0.5	2.19	0.40
	1	8.73	0,10
	1.5	30.62	0.03
G. gracilis	0	1.00	1.00
	1.5	23.54	1.59
	2	71.02	1.00
	2.5	249.76	0.40
G. corneum	0	1.00	1.00
	1	28.66	0.03
	1.5	115.16	0.001
	2	437.39	0.00002
