# Supplementary figures and images for: Three-Dimensional Printing of Red Algae Biopolymers: Effect of Locust Bean Gum on Rheology and Processability
Source: Gels. 2024 Feb 23;10(3):166. doi: 10.3390/gels10030166 (PMC10970507; doi:10.3390/gels10030166)

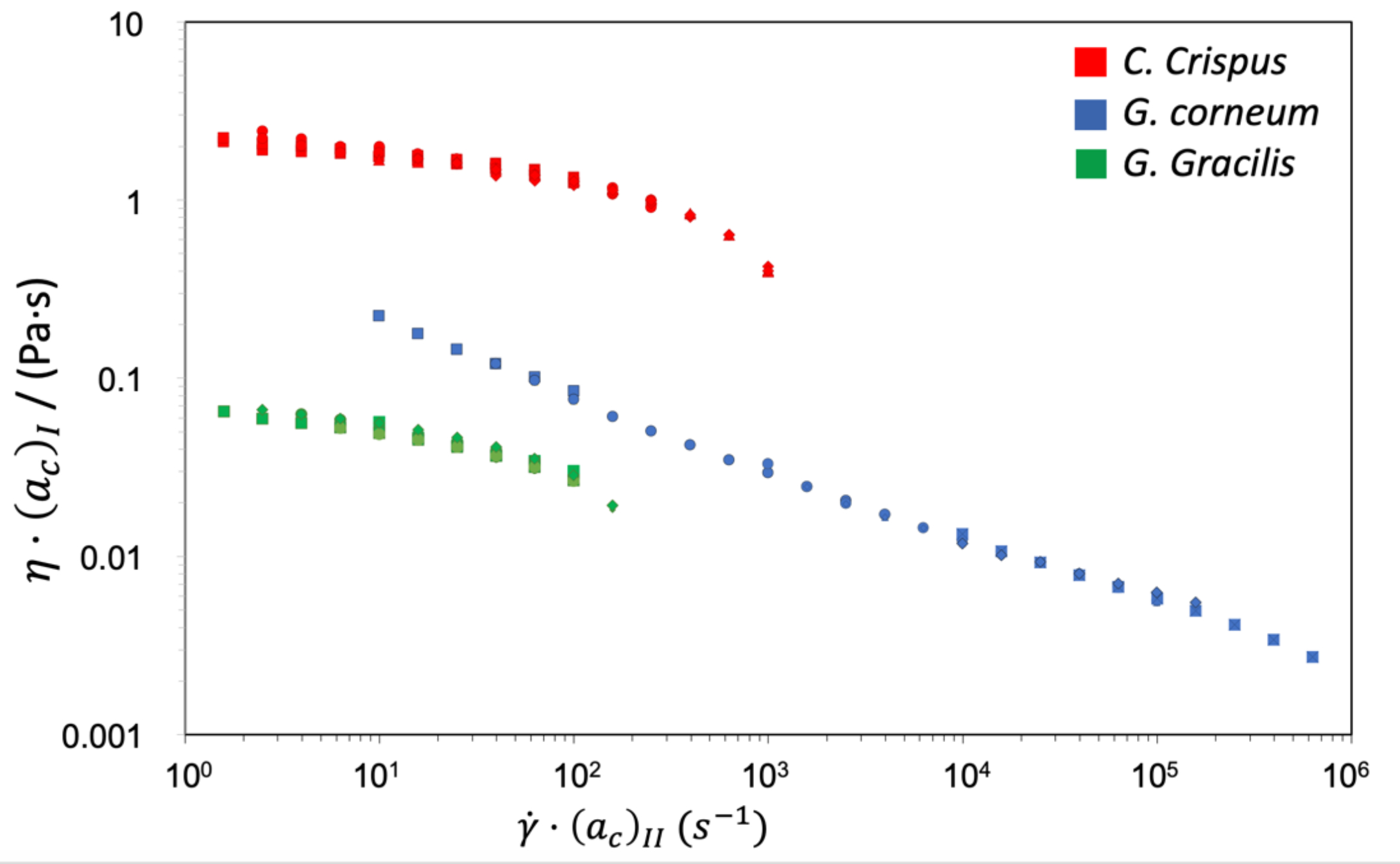

Supplement: Supplementary file 1 [file gels-10-00166-s001.zip › Supplementary Figure S1.jpg]
